# Lived experiences of Ethiopian women post-mastectomy without reconstruction: findings and guidelines from a feminist phenomenological study

**DOI:** 10.1007/s00520-026-10593-2

**Published:** 2026-03-25

**Authors:** Blen Alemu Gebremedhin, Gloria Thupayagale-Tshweneagae, Abdu Adem Yesufe

**Affiliations:** 1 Department of Health Studies, University of South Africa, Pretoria, South Africa; 2 Oncology Unit, St. Paul Hospital Millennium Medical College, Addis Ababa, Ethiopia

**Keywords:** Mastectomy, Lived experiences, Sexuality, Supportive care, Feminist phenomenology, Coping strategies

## Abstract

**Purpose:**

The lack of evidence-based knowledge about women’s experiences after mastectomy has serious implications for mastectomy-supportive care in the Ethiopian health system. Thus, the purpose of this study was to explore the lived experiences of women who had a mastectomy without reconstruction and consequently develop guidelines for healthcare providers to enhance supportive care.

**Methods:**

This qualitative study is grounded in feminist values and Heidegger’s philosophy of existential phenomenology. It was conducted at St. Paul’s Hospital Millennium Medical College, one of the largest hospitals in the country, which provides cancer care. Purposive sampling was used until saturation was reached. Thus, twenty women with an average survival time of 2 years who had undergone mastectomy without reconstruction were interviewed in-depth about their mastectomy experience. The data were analysed using interpretive phenomenological analysis (IPA) employing the ATLAS.ti 9 version qualitative data analysis software.

**Results:**

Five themes emerged from the analysis of the interview data: body image and appearance-related concerns, psychological effects and social relationships, concerns about sexual function, healthcare-related experiences, and coping strategies. The findings of this study showed that women encounter ongoing challenges following breast cancer diagnosis and mastectomy that healthcare workers do not adequately address.

**Conclusion:**

Consequently, the findings resulted in the development of guidelines to promote quality mastectomy care tailored to the needs of women who choose or are unable to undergo reconstruction. Policymakers, health care administrators, and professionals can use these guidelines as a resource for initiatives to improve supportive care for women who have had a mastectomy without reconstruction.

## Introduction

Noncommunicable diseases (NCDs) have become a significant global concern, accounting for over 70% of global mortality [1]. Among these, female breast cancer emerges as a prevalent issue, with an estimated 2.3 million new cases and 670,000 deaths annually, disproportionately affecting transitioning countries [2]. Ethiopia, characterised by rapid population growth and a high cancer burden, contributes up to 7.3% of the total national mortality rates [3, 4]. Breast cancer constitutes a significant portion of all cancer cases in Ethiopia, alongside cervical and ovarian cancers, with an age-standardised rate of 40.6 per 100,000 women [5].

Mastectomy, a common treatment for breast cancer, significantly impacts women’s physical, emotional, and psychological well-being. This surgical procedure often leads to feelings of uncertainty, powerlessness, and depression, affecting body image, self-esteem, and sexuality [6]. Physical changes such as breast asymmetry, scars, and posture alterations result in varied emotional responses—from relief to demoralisation—affecting social interactions and family dynamics [7]. Furthermore, mastectomy profoundly affects women’s self-concepts and can lead to social withdrawal, while also impacting intimacy and sexuality [8]. Effective communication between healthcare professionals and patients on post-mastectomy sexuality is crucial yet often neglected, contributing to anxiety, depression, fear, and social dysfunction among women who have had a mastectomy [9].

Unlike other cancers, breast cancer is distinct in that it is linked to external femininity, women’s sexualized bodies, and the social objectification of the female body [10]. In recent years, the *“going flat”* movement has gained attention globally, advocating for women who choose not to undergo breast reconstruction after mastectomy. This movement challenges the assumption that reconstruction is necessary to restore femininity and promotes body autonomy and acceptance [11]. In low-resource settings like Ethiopia, where reconstruction options are limited or unavailable [12] many women live with a flat chest by necessity rather than choice.

Studies emphasise the necessity of rehabilitation services for cancer patients, with health professionals’ increased awareness of patient experiences crucial for mitigating post-treatment side effects, maintaining quality of life, and improving survival rates [13]. Tailored supportive care—considering age, individual, social, and cultural contexts—aims to enhance quality of life through educational, social, psychological, and rehabilitation services. Yet, access to such care is often hindered by low prioritisation, limited resources, and insufficient data on patient needs [14, 15]. Over the years, more attention has been paid to mastectomy’s medical aspects than to its social and psychological consequences. Of greater importance is the fact that empirical information about the patient’s lived experience has benefits to healthcare providers in delivering enhanced care [16].

Despite the high incidence of breast cancer in Ethiopia, most existing literature primarily focuses on knowledge, incidence, prevention, diagnosis, and treatment [17–20]. While globally, Patient-Reported Outcome Measures (PROMs) are widely used to assess the physical, emotional, and social impact of mastectomy, these tools often rely on structured questionnaires that may not fully capture the depth of lived experiences [21]. In low-resource settings like Ethiopia, PROMs are rarely utilised, and there is a notable lack of qualitative studies exploring the post-mastectomy lived experience [22, 23]. This study seeks to address that gap by using interpretive phenomenological methods to explore how women who have undergone mastectomy without reconstruction perceive their bodies, sexuality, and social roles. These questions emerged as central due to the profound psychosocial and relational challenges reported by women in similar contexts, and the absence of culturally sensitive, patient-centred care guidelines. Specifically, the study aims to answer the following questions:

- How did women who had a mastectomy without reconstruction perceive their bodies through the experience?
- What were the effects of mastectomy on women's sexuality?
- What were the psychological effects and social relationships of women after mastectomy without reconstruction?
- What helped women who have had a mastectomy without reconstruction rationalise and continue with life?
- What key components should be included in guidelines for healthcare providers to improve supportive care for women who have had a mastectomy without reconstruction in Ethiopia?

By understanding these lived experiences, the study aims to inform the development of supportive care guidelines tailored to the Ethiopian context, ultimately enhancing the quality of life for affected women and their families.

### Theoretical framework

The study has employed feminist phenomenology to have a deeper understanding of how Ethiopian women interpret the living experience of mastectomy, to reflect on gender, cultural, and systematic oppression and its significance to women’s well-being and the possible systems needed to address mastectomy in the future. Heideggerian existential phenomenology has been used to describe women’s post-mastectomy experiences by listening to and interpreting their lived experiences, and a feminist approach was used to describe how sex and gender impact their experience and advocates for changing social, political, and health policies that affect women’s lives [24].

Feminist phenomenology and medicine bring together two components in phenomenological research. Foremost, as many feminist scholars pointed out, the philosophical tradition of phenomenology proposes important resources for addressing topics regarding the lived experience of marginalisation, invisibility, non-normativity, and oppression. Furthermore, it offers a means of developing the phenomenological structure by questioning how experiences, for instance, sexuality, sexual difference, pregnancy, birth, race, and ethnicity, inform phenomenology as a philosophical scheme. Next, phenomenological inquiries have provided an important analysis of significance for medical practice, such as analysis of an experience of illness, pain and bodily alienation, and the meaning of health. While there is a thriving field of feminist phenomenology addressing objective issues of embodiment, and phenomenologists have made valuable contributions to analysing the nature of medicine, the meaning of illness and health, and clinical practice, studies that integrate insights from both feminist phenomenology and the phenomenology of medicine are few. Feminist phenomenology and medicine demonstrate the merit of interweaving the two strands to develop more inclusive analyses of issues such as bodily self-experience, normality & deviance, self-alienation, and objectification, which are fundamental to both themes. By taking the embodiment of subjective life and experience seriously and bringing different forms of embodied existence to the description and analysis, feminist phenomenology and medicine seek to develop and sharpen phenomenology's methodological tools and conceptual framework [25].

In summary, by incorporating Heideggerian existential phenomenology, the study described the experiences of women who had a mastectomy without reconstruction and employed a feminist perspective to analyse the impact of sex and gender. The integration of feminist phenomenology and medicine aimed to enhance the understanding of embodiment, illness, and health, to improve support and care for women post-mastectomy without reconstruction. Overall, the study emphasised the importance of considering diverse perspectives and advocating for social and political changes to address the needs of these women effectively.

## Methods

This qualitative study used an interpretive phenomenology approach grounded in feminist theory. The study used phenomenological methods that led to a tradition of qualitative research rooted in the work of the philosophers Edmund Husserl (1859–1938) and Martin Heidegger (1889–1976). Phenomenological methods are useful when it comes to highlighting the experiences and perceptions of people from their viewpoints [26]. Therefore, the researcher has used phenomenological methods to effectively highlight the experiences and perceptions of women who have undergone mastectomy without reconstruction from their perspective, allowing for a deeper understanding and explanation of their lived experience.

The study was carried out at St. Paul’s Hospital Millennium Medical College, one of the largest hospitals in the country, providing cancer care. Participants were selected using purposive sampling based on specific inclusion and exclusion criteria. Women eligible for inclusion were those aged 18 years and above, fluent in spoken and understanding Amharic, and who had undergone a single or bilateral mastectomy at least one year before the study. Exclusion criteria included women who had undergone breast reconstruction surgery and those with cognitive impairments. Cognitive impairment was defined as any condition that significantly affected memory, comprehension, or communication, thereby limiting the participant’s ability to provide informed consent and articulate their lived experiences. Eligible participants were identified in collaboration with oncology department staff, who assisted in approaching potential candidates during their scheduled visits.

Informed consent was obtained before data collection. Participants were required to read and sign a consent form. In cases where participants couldn't read, the researcher read the form aloud, addressing any queries. They were briefed on confidentiality measures and their right to withdraw without affecting future care. Anonymity was preserved, and data were securely stored electronically with password protection. Ethical clearance for data collection was obtained from the University of South Africa Research Ethics Committee and St. Paul’s Hospital Millennium Medical College. Moreover, pseudonyms were assigned to all participants to ensure their anonymity and confidentiality.

Interviews were conducted in a private office within the hospital at suitable times for the participants and were conducted in Amharic, the language spoken and understood by all participants. As the researcher is the principal instrument in qualitative research [27], the researcher conducted the interviews. The researcher received training on qualitative interview skills at the University of South Africa in a workshop organised by the College of Graduates. The training emphasised techniques such as reflection, summarising, and the importance of building trust with participants by talking less and listening more.

Semi-structured in-depth interviews were conducted using a guide with a grand tour question and related thematic questions. The guide was informed by the study’s research questions, relevant literature, and the theoretical framework grounded in feminist and Heideggerian phenomenology. These themes were translated into open-ended questions designed to elicit rich, detailed narratives. The guide was structured to begin with warm-up questions to build rapport, followed by thematic questions covering body image, sexuality, social life, coping mechanisms, and healthcare experiences. Probing questions were included to deepen responses and explore sensitive areas, while closing questions allowed participants to share additional insights. A pilot test was conducted to refine the guide for clarity, relevance, and flow. The final version included an introduction, demographic prompts, thematic questions with probes, and closing remarks, ensuring a smooth and respectful interview process that respected participants’ voices and experiences.

All interviews were audio-recorded with participants’ consent to ensure accuracy and completeness of the data. The recordings were first transcribed verbatim in Amharic, the participants’ native language, to preserve the authenticity of their expressions and meanings. The transcripts were then translated into English by the researcher, who is fluent in both languages and familiar with the cultural context. This approach ensured that the nuances of participants' lived experiences were retained throughout the data analysis process.

Data were then analysed by the researcher using Interpretive Phenomenological Analysis (IPA) to explore how participants made sense of their experiences. The process began with re-reading transcripts and listening to audio recordings, followed by descriptive and conceptual observations to develop preliminary analyses. Excerpts were categorised into themes using a spot-on coding approach, and ATLAS.ti software (version 9) supported a cyclical and iterative analysis, enabling the development of code chains, thematic networks, and overarching patterns.

The researcher focused on credibility, dependability, confirmability, transferability, and authenticity to ensure trustworthiness. Credibility was achieved through detailed explanations and rapport-building during interviews. Dependability involved a well-documented process with audio-recorded and transcribed interviews. Confirmability was ensured by representing participants' responses without bias and maintaining transparency. To enhance confirmability and authenticity, the researcher engaged in ongoing reflexivity throughout the study. This included maintaining a reflexive journal to critically examine personal assumptions, emotional responses, and interactions with participants. As an Ethiopian woman with professional experience in health and social care, the researcher acknowledged her positionality and its potential influence on data interpretation. This reflexive practice helped ensure that the findings remained grounded in participants lived experiences rather than the researcher’s preconceptions. Transferability was enhanced through meticulous transcription and reflection on biases. Authenticity was achieved by faithfully capturing participants’ experiences and emotions.

## Results

Twenty women, aged between 28 and 62, participated in the in-depth interviews for the study. All these women had undergone unilateral mastectomy surgery without reconstruction, with an average survival time of around 2 years. Most participants (80%) were married. The results indicated that living with mastectomy without reconstruction provided different experiences related to physical, psychological, sexual and social aspects. These experiences were summarised into five themes below:

### Body image and appearance-related concerns

For many women, the loss of a breast created a feeling of not being a woman anymore and impacted their sense of identity. The societal objectification of the female body and the association of a woman's worth with her physical appearance and sexual function intensified the pressures faced by women who have had a mastectomy.

Participants revealed significant body-related distress and dissatisfaction. For instance, Melat shared, *“I hated my body and everything about myself,”* expressing a deep rejection of her altered physical self. Netsanet similarly described a visceral aversion to her body: *“I do not want to see it in the first place. When I see it, I become nauseated and feel like I am about to pass out. Even when I get dressed, I become extremely sick.”* This distress led to a reluctance to engage in social activities. *“I do not want to go anywhere,”* shared Halima, reflecting a common sentiment among the participants. Melat acknowledged feeling constrained, stating, *“There is something that holds you back.”* At the same time, Hirut emotionally recounted declining a wedding invite due to self-consciousness, admitting, *“I could not possibly attend a wedding with one flat chest.”*

Emotions of embarrassment and scrutiny were prevalent, with Hanan describing the sensation of being talked about while in public spaces. *“As you walk down the street and see people talking, you might think they're talking about you,”* she expressed. Within the confines of their homes, changing clothes in front of family members brought about discomfort. Hanan articulated her reservations, saying, *“I do not want my kids to see me… Even in my compound, I do not want to go out without a scarf.”*

In Ethiopia, breast reconstruction surgery is not common, and there are no commercially available brassieres for women who have had a mastectomy, which exacerbates challenges related to clothing and appearance. Participants had difficulties finding clothes that looked good on them and sought ways to hide the asymmetry caused by surgery. Meg deliberately covered her chest with fabric, sharing, *“I purposefully covered it even when I was in our compound because the tenants are unaware of my condition.”*

However, some participants highlighted the priority of survival over body image concerns. Genet emphasised, *“Nothing is more important than my health,”* while Feruza stressed, *“The most important thing is that I survive.”*

### Psychological effects and social relationships

Women’s responses to mastectomy varied greatly, with some feeling shocked, disturbed, panicked, and frightened, while others experienced feelings of hopelessness. One participant described it as feeling *“as if someone told me of my death”* (Abeba), while others expressed feeling drained, mentally strained, and physically incapacitated. Throughout discussions, participants often reflected on how their mastectomy experience had changed them, either for the better or worse. Many noted a significant loss of energy, heightened irritability, and an increased sensitivity to everyday noises. Abeba articulated this sensitivity, stating, *“A sound itself disturbs you… Even my children’s voice itself disturbs me… I do not feel like a normal person.”*

The emotional impact of mastectomy extends to concerns about motherhood roles and the effects on their families. Participants express fears about not being present to witness their children's growth and feelings of sadness and guilt for potentially not fulfilling their maternal duties. Feruza shares deep sorrow for her daughter’s future, saying, *“I feel sorry for my daughter… I am not sure who I should leave my child with.”* Bezunesh reflects on her struggles, stating, *“It is better if I do not remember it… I hate everyone, including my baby, as I could not even hold her.”*

Balancing illness and childcare leads to guilt, with participants expressing inadequacy in shouldering the family’s burdens. Concerns about burdening loved ones also result in emotional suppression. Semira shares her worries, *“If I were weak and always in bed, I am afraid my children would get tired of me.”* Despite these challenges, love for their families serves as a source of resilience. Melat draws strength from her child, saying, *“I have a child, and when I think of him, I want to live… My children were the ones who detained me.”*

The disruption of regular routines following mastectomy often resulted in the neglect of hobbies and social connections. Many women preferred solitude, avoiding social interactions due to a fear of judgment and negative societal perceptions. Melat described this stigma, *“Our people believe you have done something wrong and have been punished by God.”*

The impact of the COVID-19 pandemic and ensuing social distancing measures further exacerbated these feelings of isolation, leaving women yearning for normal social interactions. Returning to work presented challenges, with participants feeling self-conscious and uneasy around colleagues, saying, *“I am afraid when my colleagues look at me. So, I arrive at my office early in the morning and leave my workplace after everyone has left… I am not comfortable socialising yet.”*

Despite the difficulties, maintaining a social life was seen as crucial for coping and well-being, providing a sense of normalcy and equality. A few participants chose to be open about their experiences and found support from others. One participant even expressed how her experience motivated her to socialise more and participate in social activities, stating, *“Even in the past, my social life was weak. I have become good now.”* (Aster).

### Concerns about sexual function

Post-treatment, participants reported various sexual difficulties stemming from the physical, psychosocial, and cultural impacts of mastectomy on their sexuality. Abeba expressed, *“This cancer not only finished my hair and other things, but I think it did the same with my sexual desire.”* Although the decreased desire may not be noticeable from their partners’ perspective, some women reported a decline in their spouse's sexual desire after the surgery. Nejat mentioned, *“He is no longer interested in touching me… he might assume I do not want to have sex; therefore, he is trying to be considerate of my feelings… but I believe he despises seeing the scar when we do it.”* Abebech expressed her concern, saying, *“Even though it has been two years since my surgery, we did not do it… I believe his love for me has ended.”*

In the study discussion, only six out of fifteen participants with available partners engaged in sexual activity. Many women felt obligated to participate in sex due to marital duties or religious beliefs rather than deriving pleasure from it. Hanan emphasised fulfilling her husband's needs in line with Islamic teachings, stating, *“If you make your husband happy, God will make you happy.”* Abeba also mentioned meeting her husband's needs to prevent his potential infidelity despite her lack of personal interest and unhappiness.

The fear of sexual rejection and societal pressure to maintain marital harmony often prevented these women from expressing their own needs or seeking help to enhance their sexual experiences. This taboo extended to healthcare providers, leading to limited and non-need-oriented sexual communication with these women.

Melat chose to abstain from sexual activity post-surgery, perceiving it as harmful to her health. She even considered divorce if her husband did not respect her decision, expressing disappointment in his lack of empathy and questioning the necessity of pleasing him when her needs were disregarded.

The shift in sexual desire and behaviour significantly impacted marital relationships, either exacerbating existing issues or creating new ones. Some women contemplated divorce, while in contrast, there were instances where couples demonstrated understanding and care for each other's feelings post-surgery. Certain husbands remained supportive and attentive towards their wives.

### Healthcare-related experience

Most women who have had a mastectomy expressed gratitude towards healthcare workers for their physical and psychological care. They appreciated doctors and nurses for their kindness and professionalism. Hanan mentioned, *“When that doctor came near me, he calmed my heart… Some doctors treat more than medicine.”* Despite occasional impropriety, participants understood the heavy workload of healthcare workers and expressed appreciation for their efforts. Nejat noted, *“There are unpleasant nurses, and there are nice nurses that treat me well and make me happy when I see them, so it is a mix.”*

However, some women were dissatisfied with inappropriate approaches and neglect from healthcare workers. Additionally, prejudice from male healthcare workers added pressure on female patients. While most male health professionals were professional, a few made extreme comments that left the women feeling vulnerable and powerless. Halima shared, *“That doctor is always very irritated… If you go in there, you will probably urinate on your pants cause of anxiety… If you say something wrong, you must deal with what comes down to you… He is a truly angry man… You cannot say a word there… You just stand there like a statue.”* Senait was also deeply saddened by an unpleasant remark from her doctor when she asked if she could give birth after treatment, hoping for guidance. The doctor responded, *“This question is not expected from you. Let me tell you what it is like to have a baby. It is like pooping on an asphalt road.”*

Instances of inadequate communication and negligence were also reported. Tenagne, who had surgery at a different hospital but a follow-up at the study hospital, sadly recounted that she was unaware her entire breast had been removed. The doctor had informed her only the tumour would be removed, and she discovered the change in her surgery herself three days later. Additionally, Abeba highlighted inconsistent blood pressure measurements and the failure to address her hypertension history during chemotherapy sessions, which raised concerns about her health.

Moreover, the physical setting of the facility also negatively impacted patients' experiences. Abeba highlighted the need for a functional television in the chemo room: *“The chemo room has a television, but most of the time, it is broken.”* Similarly, Meg expressed dissatisfaction with the environment and infrastructure: *“You go well, and you will be depressed there… The environment you see is not good… There is also a strange noise… Crowded there…”.*

Participants reported a lack of regular psychological counselling during their treatment, with doctors providing technical support but lacking guidance on coping with emotional and practical challenges. When available, counselling often focuses on specific topics like family planning rather than broader concerns such as sexuality. Furthermore, counselling was primarily provided during chemotherapy sessions, despite the need for support at every stage of treatment. Additionally, many women expressed the need for guidance on nutrition and education on preventing cancer relapse. The need for counselling also extended to partners, families, and caregivers, especially in communities with limited knowledge about cancer.

The cost of breast cancer treatment poses a significant burden for many women, including expenses related to surgery, chemotherapy, diagnostic tests, prescription drugs, and other medical costs. Financial concerns often create stress for these patients. For example, Semira expressed her overwhelming experience with the cost: *“I was overwhelmed by the cost of the treatment. My children are the ones who made me get treated … My daughter even sold her gold to pay for my treatment, and she lost her fortune.”*

Women dependent on men for financial support face significant challenges in getting the care they need. Melat shared her struggle when her husband refused to pay for her medication: *“There is an argument over when I said ‘I ran out of pills’ … We got to the point where he would not buy it when there were just two or three rounds of treatment remaining.”*

Having health insurance has proven beneficial for some women, but it is not accessible to everyone. The lack of availability of certain drugs and procedures in public health institutions further contributes to the financial burden.

### Coping strategies

Family support is crucial for women undergoing breast cancer, with many appreciating the sacrifices and emotional backing from their loved ones. Conversely, the absence of such support can be devastating, emphasising the importance of assistance from friends, neighbours, and community networks for emotional and financial aid.

Connecting with other women who have faced similar challenges is highly valued. Hearing stories of survival and witnessing others leading fulfilling lives post-mastectomy instils hope and motivation. However, survivors must share their experiences positively, as negative narratives can infuse fear and discouragement. Breast cancer support groups, like the *Pink Lotus Ethiopia Support Group*, provide a vital platform for women to connect and share experiences. Social media and the media also play key roles in disseminating information and stories of mastectomy survivors. On the other hand, Aster highlighted the need for the media to focus on positive narratives of cancer patients to spread messages of hope, stating, *“These artists always make dramas which show a cancer patient’s death … (laughs) … That ought not to be the case. Cancer patients can survive, and this should be emphasised in their dramas.”*

Furthermore, the study participants highlighted the crucial role of their faith in coping with breast cancer and mastectomy, attributing their journey and survival to the strength and encouragement derived from their belief in God. They trusted in God’s healing power, viewing their bodies as gifts from Him and finding solace in the understanding that He has the right to take what He gives. Religious practices such as personal prayer, familial and religious communal prayers, reading the Bible, listening to sermons, meditating, and engaging in salat (prayer) were mentioned as sources of comfort and encouragement. While most participants found spiritual solace, one individual experienced a weakening of spirituality post-surgery but sought comfort in God’s promises during moments of struggle.

Some participants in coping with cancer expressed the significance of their willpower and determination to survive, while for others, their motivation to persevere came from the essence of living for their loved ones. Additionally, a few women exhibited a competitive spirit derived from their life experiences. For example, Seada, having learned from life’s challenges, expressed fearlessness, saying, *“Life has taught me a lot… I believe that everything will work out.”*

## Discussions

Mastectomy poses significant body image and appearance concerns for women. In Ethiopia, despite advancements in less disfiguring breast cancer treatments globally [28], the standard remains a modified radical mastectomy for stages 1 to 3 [29]. This contrasts with historical Western breast cancer activism that challenged the patriarchal radical mastectomy approach, evolving into modern advocacy for more favourable treatment choices and collaborative research efforts [30, 31].

Following mastectomy, many women reported discomfort with their appearance, leading to social withdrawal and reluctance to attend public gatherings. To manage asymmetry, participants improvised with garments under their brassieres, a practice also observed among Ghanaian women due to limited access to reconstruction [32]. Although custom-made brassieres were provided at the study hospital, distribution was limited. However, feminist scholars critique the pressure to undergo reconstruction as a means of restoring femininity [33], and studies show that reconstruction does not always improve the quality of life [34]. While some women choose to remain flat to avoid further surgeries [33], others, such as Nigerian women, expressed interest in reconstruction when informed of its benefits [35]. These findings underscore the importance of informed choice and access to both reconstruction and prosthetic options.

In cultures where women are objectified, the loss of a breast can profoundly impact a woman's sense of self, femininity, and identity. Studies have shown that women often equate losing a breast with losing their womanhood and completeness, highlighting the strong connection between their bodies and self-perception [36, 37]. This loss can lead to elevated levels of anxiety, stress, depression, and other negative emotions, compounded by societal expectations that place additional pressures on women as wives, mothers, and caregivers [38].

In this study and other analyses, mastectomy often leads women to limit social interactions and adopt a constrained lifestyle, driven by concerns such as wound healing, weakened immunity, and the avoidance of social sympathy [39, 40]. Stigma and misconceptions surrounding cancer can lead women to conceal their surgery, facing blame and social isolation within their communities [32, 41].

Following breast cancer treatment, many women in formal employment cease working, with studies indicating a heightened risk of job loss for breast cancer survivors [42]. Reintegration into the workplace poses challenges, including physical and cognitive hurdles and navigating interactions with colleagues, which can either facilitate or impede the return-to-work process [43].

In this study, mastectomy impacted women’s sexual desire, often due to treatment side effects and body image issues. Husbands of women with breast cancer may also experience decreased desire because of aesthetic and cultural concerns [44]. Post-mastectomy, women may engage in sexual acts to please partners and fulfil religious expectations despite a loss of libido, underscoring the neglect of their sexual needs and reinforcing power imbalances in patriarchal family dynamics [45, 46].

This study highlights that changes in sexual desire post-mastectomy can significantly strain marital relationships. Similarly, in societies such as Pakistan, where marital contentment is intricately linked to sexual satisfaction, these changes can affect relationships [47]. The elevated divorce rates among breast cancer survivors highlight the urgent need for comprehensive support for women and their partners navigating post-treatment challenges [48]. Furthermore, the findings suggest that limited communication about sexuality was common among couples, highlighting the need for open and honest discussions. This communication gap may contribute to sexual dissatisfaction and emotional distress, as reflected in several participants’ accounts.

A minority of study participants expressed dissatisfaction with the care provided at the health facility, attributing occasional improper behaviour and neglect by healthcare providers to their heavy workloads. Additionally, the poor physical setting of the healthcare facility was cited as a source of dissatisfaction. Organisational factors, such as comfortably designed spaces, were highlighted as important for satisfactory care [49]. The existence of dominant physicians, influenced by gender and power dynamics, was also reported. In many developing countries, patriarchal norms contribute to a significant distance between patients and healthcare providers, hindering effective information sharing and limiting access to essential health information [50].

Social support is a crucial coping strategy for managing challenges linked to breast cancer treatment. Studies have demonstrated its ability to alleviate anxiety, stress, and fear while enhancing the overall well-being of patients [51–54]. On the other hand, many women utilised their faith and engaged in spiritual practices to cope with breast cancer, highlighting the essential role of spiritual beliefs in coping strategies. Some, however, struggled with feelings of confusion or anger towards God, viewing cancer as a test or punishment [55, 56].

In line with prior research [52, 57, 58], a few women leveraged their strong faith, life experiences, stress-resilient nature, and commitment to loved ones to confront and navigate the challenges posed by breast cancer.

These findings underscore the need for a structured, patient-centred approach to supportive care. Drawing directly from the lived experiences of participants, the following recommendations were developed to guide healthcare providers in addressing the psychosocial, clinical, and systemic challenges faced by women post-mastectomy in Ethiopia.

The development of the guideline encompasses five major areas to provide women-centred care throughout the mastectomy journey: preoperative patient education and preparedness, psychosocial support and counselling, healthcare system interventions, coping interventions, and surveillance of recurrence and new cancers. These guidelines emphasised educating women about the surgical procedure, offering empathetic and compassionate support, improving healthcare systems for timely access to care, implementing coping mechanisms for stress and anxiety, and ensuring regular monitoring for early detection of recurrence. A summary of these intervention guidelines is presented in Table 1. By focusing on these areas, the guidelines aim to enhance the quality of supportive care, address the unique needs of women who have had a mastectomy, and improve their overall well-being and quality of life in Ethiopia.

**Table 1:** Summary of intervention guidelines for the provision of women-centred mastectomy care in Ethiopian public health facilities

Guidelines	Intervention
Preoperative patient education and patient preparedness	- Ensure that the patient is aware of her condition and the available treatment options by outlining the diagnosis, possible courses of action, and side effects
	- Explain the proposed course of treatment, why it is needed, and the expected outcome
	- Ask how much detail she would like to know about the procedure before explaining it
	- Access resources and guide the patient toward proper learning resources and facilities,
	- Discuss the type of anaesthesia planned for the procedure while explaining medical terms and avoiding medical jargon
	- Inform and prepare the patient for the change and other consequences
	- Inform the patient about the postoperative recovery process
	- Increase patients’ involvement in the decision-making process
	- Have preoperative communication in a quiet and private environment
	- Facilitate experience-sharing sessions for patients with women of similar experience
	- Ensure continuity of care
Providing psychosocial support and counselling	
Body image concerns	- Prepare the women for the post-mastectomy mirror-viewing experience
	- Assess for patient body image/appearance-related concerns and explore sensitively how the woman is coping with her altered appearance
	- Offer the option of adaptive devices (e.g. breast prostheses) and/or surgery when appropriate. However, this support should not be aimed at making women aesthetically appealing as quickly as possible not strengthening the idea that women are the subject of the social gaze, it should be based on women's preferences and interests
	- Help these women know their bodies and understand that change is desirable only when they feel it is necessary, not the people around them
	- Promote positive body image and boost the patients’ self-love and acceptance
	- Help women who have had a mastectomy feel more confident about their bodies
	- Enhance the self-esteem of women who have undergone a mastectomy through physical activity therapies
	- Offer cognitive behavioural counselling to women with breast cancer about body image alterations and treatment decisions
	- In more severe cases, refer the women to a trained specialist for further interventions
	- Advocate for the initiatives of reconstruction surgery upon interest and other less figuring treatment options
	- Develop strategies to revise the social representation of the female body and the sexualisation of female breasts
Sexuality concerns	- Take the initiative to discuss the subject of sexuality (alone or with their partner)
	- Make the patient feel safe and comfortable by providing an appropriate space to discuss sexual concerns
	- Educate about the sexual sequelae of the treatment and ask about the sexual adjustment of the patients
	- Encourage open discussion and skilful communication to make it easier for women to disclose their sexual concerns, considering religious and cultural differences
	- Provide personal and/or couple counselling for women with sexual difficulties
	- Provide symptomatic treatments and medications
	- Involve partners in the treatment process
	- Should correct misconceptions about sexual health
	- Integrate sexual health education into the hospital environment by making information available in multiple formats
	- Refer for psychoeducational support, sexual counselling, marital counselling, or intensive psychotherapy, when appropriate
	- Counsel women to engage in regular physical activity consistently
Emotional distress	- Let clients know that stress is natural and should be expected and that stressful situations can arise throughout transitions
	- Assess if the woman is experiencing emotional disturbances
	- Provide an appropriate intervention and counselling service for women experiencing adjustment problems
	- Assess further if the woman is at higher risk of emotional distress
	- Refer to a psychiatrist or clinical psychologist if further intervention is needed
	- Facilitate radiotherapy services to those who need them to minimise the spread of concern and recurrence
Interpersonal concerns	- Support breast cancer patients to determine what and how to tell their children or family about their diagnosis
	- Assist women who have had a mastectomy in maintaining a balance between self-care and other maternal responsibilities
	- Encourage women who have had a mastectomy to socialise with women with similar experiences/support groups
	- Establish different platforms for different social engaging activities
	- Establish a professional-led support group to decrease feelings of social isolation
	- Encourage the inclusion of family members/caregivers in the care and support of women who have had a mastectomy
	- Offer family members/caretakers the opportunity to discuss concerns with the health care workers or refer to an appropriate counsellor
	- Develop strategies to change incorrect societal assumptions and public views on breast cancer
Healthcare-related interventions	
Experience with healthcare workers	- Create an open give-and-take relationship atmosphere
	- Build trust
	- Assess the information needs of the patient related to breast cancer and its treatment and side effects
	- Share lab results, explain medical terms, and provide patients with the information they need
	- Encourage women with breast cancer to take an active position in their health
	- Help women who have had a mastectomy to discover the tools they need to empower themselves and focus their care and treatment on only themselves and their needs
	- Take the initiative and show an interest in starting conversations about psychosocial issues with patients
	- Overcoming communication problems and enhancing physician-initiated communications
Counselling	- Consider the patient's problem normal and healthy, not an abnormal or pathological component of the cancer experience
	- Establish a favourable setting for consultation and schedule a suitable time for the counselling session
	- Create a hopeful environment that is open to possibilities instead of focusing on negative past experiences
	- Adopt a collaborative rather than prescriptive attitude by encouraging patients to access internal resources and develop their problem-solving skills
	- Design their strategy so that it emphasises patient empowerment and self-reclamation over problem-solving
	- Encourage patients to develop their problem-solving techniques rather than prescribing a single problem-solving path that all patients must follow
	- Offer counselling services on subjects that concern the patient
	- Involve the patient's partner, family, or significant other in counselling sessions as appropriate
Medical cost burden	- Advocate the inclusion of breast cancer care in social and community-based health insurance systems
	- Minimise out-of-pocket costs for breast cancer patients
	- Offer guidance or financial counselling throughout the diagnosis and treatment of breast cancer
	- Develop financial assistance programs for economically disadvantaged women with breast cancer
	- Advocate employment retention policies and medical leave for breast cancer patients
Coping interventions	- Provide emotional support and interpersonal engagement to people dealing with illness
	- Facilitate the formation of breast cancer support groups
	- Assist women seeking spiritual support in obtaining it
	- Increasing knowledge on coping with mastectomy, either independently or in addition to the provision of medical information
	- Improving coping skills through cognitive-behavioural therapy (psychotherapy or cognitive behavioural interactions)
	- Access resources and guide the patient toward complementary therapies (prayer, Yoga, massage, exercise, art, music, and dance therapy)
Surveillance of recurrence and new cancers	- Should individualise clinical follow-up care provided to breast cancer survivors based on age, specific diagnosis, and treatment protocol
	- Should ensure patients have a detailed cancer-related history and physical examination every 3–6 months for the first 3 years after first-line treatment, every 6–12 months for the next 2 years, and every year thereafter
	- Perform regular medical history and physical examination of all breast cancer survivors
	- Should refer women who have undergone a unilateral mastectomy for annual mammography on the intact breast
	- Inform and advise all women on the warning signs and symptoms of a local or regional recurrence
	- Should assess the patient’s cancer family history
	- Should screen for other cancers, as they would for patients in the general population

Taken together, this study contributes new insights by exploring the lived experiences of Ethiopian women who underwent mastectomy without reconstruction. This population has been largely overlooked in both local and global literature. Unlike previous studies that focus primarily on clinical outcomes or structured PROMs, this research uses feminist phenomenology to foreground women’s voices and contextual realities. It reveals how cultural expectations, limited access to reconstruction, and gendered healthcare dynamics shape post-mastectomy experiences in Ethiopia. The development of context-specific supportive care guidelines—grounded in these lived experiences—adds a practical dimension to the literature, offering actionable recommendations that address gaps in psychosocial, sexual, and systemic support. These findings complement and extend existing research from other low-resource settings and contribute context-specific insights that are often missing from global breast cancer literature. By centering Ethiopian women’s voices, this study adds depth to our understanding of post-mastectomy experiences and highlights the importance of culturally responsive care.

## Conclusion and recommendations

This study examined the experiences of Ethiopian women following mastectomy without reconstruction, highlighting challenges in body image, psychological well-being, sexual health, and coping strategies. It identified a lack of adequate healthcare support for these women, emphasising the need for customised guidelines and initiatives to improve care standards. To address this gap, healthcare providers should receive comprehensive training to meet the diverse needs of women who have undergone mastectomy without reconstruction. Specialised support programs, including psychosocial services and sexual health education, should be developed. Public awareness campaigns can help reduce stigma, while improving healthcare infrastructure is crucial for ensuring equitable access to quality care. Collaboration among healthcare professionals, support groups, and stakeholders is key to delivering comprehensive care. Implementing these strategies can enhance the Ethiopian healthcare system’s ability to provide patient-centred support and improve the well-being of women post-mastectomy without reconstruction.

### Strengths and limitations of the study

This study marks a significant milestone in Ethiopia, employing a feminist phenomenological lens to explore the experiences of women who had a mastectomy without reconstruction, providing unique perspectives for healthcare providers. The qualitative methodology used enriched the research by gathering a wealth of detailed data, allowing participants to extensively share their experiences. However, the study’s strength is accompanied by limitations. The sample size of twenty women may not fully capture the diversity of women post-mastectomy without reconstruction in Ethiopia, potentially limiting the generalizability of the findings. The voluntary nature of participation could introduce bias, and the scarcity of regional literature hindered the full validation of the study against existing research in Africa.

## Data Availability

No datasets were generated or analysed during the current study.
